# Evaluating Serum Heat Shock Protein Levels as Novel Biomarkers for Atrial Fibrillation

**DOI:** 10.3390/cells9092105

**Published:** 2020-09-16

**Authors:** Denise M. S. van Marion, Eva A. H. Lanters, Kennedy S. Ramos, Jin Li, Marit Wiersma, Luciënne Baks-te Bulte, Agnes J. Q. M. Muskens, Eric Boersma, Natasja M. S. de Groot, Bianca J. J. M. Brundel

**Affiliations:** 1 Department of Physiology, Amsterdam Cardiovascular Sciences, Amsterdam UMC, Vrije University, 1081HV Amsterdam, The Netherlands; d.vanmarion@amsterdamumc.nl (D.M.S.v.M.); k.silvaramos@amsterdamumc.nl (K.S.R.); j.li@amsterdamumc.nl (J.L.); m.wiersma1@amsterdamumc.nl (M.W.); lucienne.baks@gmail.com (L.B.-t.B.); 2 Department of Cardiology, Erasmus MC, 3000CA Rotterdam, The Netherlands; Eva_Lanters@hotmail.com (E.A.H.L.); a.muskens@erasmusmc.nl (A.J.Q.M.M.); h.boersma@erasmusmc.nl (E.B.); n.m.s.degroot@erasmusmc.nl (N.M.S.d.G.); 3 Netherlands Heart Institute, 3511EP Utrecht, The Netherlands

**Keywords:** atrial fibrillation, heat shock protein, electrical cardioversion, pulmonary vein isolation, biomarkers

## Abstract

**Background:** Staging of atrial fibrillation (AF) is essential to understanding disease progression and the accompanied increase in therapy failure. Blood-based heat shock protein (HSP) levels may enable staging of AF and the identification of patients with higher risk for AF recurrence after treatment. **Objective:** This study evaluates the relationship between serum HSP levels, presence of AF, AF stage and AF recurrence following electrocardioversion (ECV) or pulmonary vein isolation (PVI). **Methods:** To determine HSP27, HSP70, cardiovascular (cv)HSP and HSP60 levels, serum samples were collected from control patients without AF and patients with paroxysmal atrial fibrillation (PAF), persistent (PeAF) and longstanding persistent (LSPeAF) AF, presenting for ECV or PVI, prior to intervention and at 3-, 6- and 12-months post-PVI. **Results:** The study population (*n* = 297) consisted of 98 control and 199 AF patients admitted for ECV (*n* = 98) or PVI (*n* = 101). HSP27, HSP70, cvHSP and HSP60 serum levels did not differ between patients without or with PAF, PeAF or LSPeAF. Additionally, baseline HSP levels did not correlate with AF recurrence after ECV or PVI. However, in AF patients with AF recurrence, HSP27 levels were significantly elevated post-PVI relative to baseline, compared to patients without recurrence. **Conclusions:** No association was observed between baseline HSP levels and the presence of AF, AF stage or AF recurrence. However, HSP27 levels were increased in serum samples of patients with AF recurrence within one year after PVI, suggesting that HSP27 levels may predict recurrence of AF after ablative therapy.

## 1. Introduction

Atrial fibrillation (AF) is the most common cardiac arrhythmia, with a rising prevalence due to the aging population [1]. Proper staging of AF is essential to select the optimal treatment strategies to prevent AF progression, and the accompanied risk to develop severe complications such as thromboembolic events, heart failure, cognitive impairment and increased mortality [2,3]. At present, AF can only be diagnosed with a surface electrocardiogram when a patient already suffers from AF, presenting with palpitations or thromboembolic complications. However, diagnosis of AF may be challenging in patients with asymptomatic or very short-lasting episodes of AF [4]. In addition, treatment aimed at rhythm control, such as ablative therapy, is less successful in patients with persistent AF, compared to paroxysmal AF [3]. Hence, proper staging of AF and the start of effective treatment of AF is of utmost importance. Therefore, there is an urgent need to identify diagnostic biomarkers to stage AF and guide patient-tailored therapy [5].

Biomarkers are widely accepted as a diagnostic tool to screen or monitor patients for a variety of cardiovascular diseases. At present, there are no recommendations on the use of AF-specific biomarkers in the most recent guidelines [3,6], despite the fact that several blood-based biomarkers related to AF pathology have been identified. These biomarkers include brain natriuretic peptides, cancer-antigen-125, fibroblast growth factor-23 [7,8] and -21 [9], highly sensitive cardiac troponin I [10], homocysteine [11], (a)symmetric dimetylarginine [12], interleukine-6 and matrix metalloproteinase-9/tissue inhibitor of metalloproteinase-1 ratio [13]. Although potential AF-related biomarkers are available, the role of these biomarkers in staging of AF (paroxysmal or (longstanding) persistent AF) or predicting AF recurrence after AF therapy has only be moderately studied.

Emerging evidence indicates that heat shock proteins (HSPs) may represent a suitable biomarker to predict AF recurrence after treatment. HSPs are chaperones that play an important role in safeguarding proteostasis, the homeostasis of protein expression, and function and degradation in cells [14]. The derailment of proteostasis has been identified as a key factor underlying electropathology and AF progression [14,15,16]. During stress or disease, such as AF, especially, activation of the heat shock transcription factor 1 regulates HSP transcription [17]. Within the HSP family, small HSPs, including HSP27, are probably the most important in maintaining proteostasis in cardiomyocytes by stabilizing the contractile proteins [18,19,20,21]. Previously, atrial HSP27 levels were found to be induced in atrial tissue samples of patients with paroxysmal AF, while tissue HSP27 levels get exhausted in patients with (longstanding) persistent AF [18], indicating that low tissue HSP levels are associated with AF progression. The study of Hu et al. described that low baseline serum HSP27 levels of patients who received ablative therapy predict AF recurrence and patients with high baseline serum levels of HSP27 showed an improved maintenance rate of sinus rhythm [22]. So far, it is unknown which HSP family member(s) represent biomarkers to identify the stage of AF and recurrence after therapy. In the current study, various members of the HSP family, including HSP27, HSP70, cardiovascular (cv)HSP and HSP60, were measured in serum samples of control and patients with paroxysmal atrial fibrillation (PAF), persistent atrial fibrillation (PeAF) and longstanding persistent atrial fibrillation (LSPeAF), undergoing elective electrical cardioversion (ECV) or pulmonary vein isolation (PVI), to identify whether HSPs associate with the stage of AF and recurrences after either PVI or ECV. Herein, we report that baseline HSP levels between control and AF patients are comparable. However, HSP27 levels were increased in follow-up samples of patients with AF recurrence after PVI, suggesting that increased HSP27 levels may predict recurrence of AF after ablative therapy.

## 2. Materials and Methods

### 2.1. Study Popultion

From December 2014 till November 2016, 297 subjects >18 years with or without a history of AF were prospectively enrolled for the HALT & REVERSE study [23] at the department of cardiology in the Erasmus MC, Rotterdam, the Netherlands. The study population consists of a control group of patients without a history of AF and a study group of patients with symptomatic AF who were scheduled for electrical cardioversion (ECV) or pulmonary vein isolation (PVI).

The control group consisted of patients (*n* = 98) who were scheduled for elective ablation of premature ventricular contractions (PVC), Wolff–Parkinson–White syndrome (WPW) or Ajmaline testing. These patients were eligible for inclusion in case of absence of structural heart disease and any atrial tachyarrhythmia. Blood serum samples were obtained 1 day prior to the scheduled intervention. Follow-up was not performed.

The study group included patients presenting for ECV (*n* = 98) or PVI (*n* = 101) for either symptomatic PAF (less than 7 days of AF), PeAF (having AF between 7 days and 1 year) or LSPeAF (more than 1 year of AF). Exclusion criteria included paced atrial rhythms, cancer, inflammation and rheumatic diseases. Blood samples were obtained 1 day prior to the scheduled intervention. PVI was unsuccessful in two patients.

Patients who underwent PVI visited the outpatient clinic at 3-, 6- and 12-months after the procedure to provide follow-up serum samples and to screen for AF recurrences. Due to rescheduling of patients to different hospitals, follow-up serum samples were not available for some patients in both the AF recurrence and no recurrence group. In addition, AF recurrence data is available via recordings of inbetween visits due to AF complaints of the patients. AF recurrence was defined as an AF episode documented on either a 12-lead surface ECG or Holter monitor recordings. In patients undergoing ECV, for each individual patient only 3-, 6- and 12-months post-procedural follow-up telephone consultations were scheduled. The study endpoint was the completion of the 12-months follow-up period or earlier due to withdrawn informed consent, pacemaker implant or AF recurrence. Clinical characteristics were obtained from the electronic patients’ files.

All patients signed written informed consent prior to inclusion. This sub-study is part of the HALT AND REVERSE trial (MEC-2014-393), and is approved by the institutional medical ethical committee. The study is carried out according to the principals of the Declaration of Helsinki in accordance with the Medical Research Committee involving the Human Subjects Act.

### 2.2. HSP Measurement in Serum Samples

Immediately after blood sample collection, serum was harvested from blood in BD Vacutainer™ SST™ II Advance Tubes (Fisher Scientific, Bleiswijk, the Netherlands) by centrifugation at 2000× *g* for 10 min at 4 °C and frozen in −80 °C until analysis of HSP27, HSP70, cvHSP and HSP60 levels. For measurement of serum HSP27 levels, samples were diluted six times, and for HSP70 levels samples were diluted twice in 1% BSA in PBS. The amount of HSP27 and HSP70 protein was detected in triplicates using human HSP27 or HSP70 DuoSet^®^ ELISA kits from R&D (Cat. no. DY1580 and DY1663, respectively, Minneapolis, MN, USA) according to the manufacturer’s instructions with minor adjustments (serum was incubated at 4 °C overnight, instead of 2 h at room temperature). Undiluted serum was used to measure cvHSP protein (singular) with ELISA kits from Cusabio (CSB-EL010838HU, Houston, TX, USA), according to manufacturer’s instructions with minor adjustments (kept incubation temperature at 20 °C). Undiluted serum was used to measure HSP60 protein in duplicate with the HSP60 DuoSet^®^ ELISA kit from R&D (DYC1800), according to manufacturer’s instructions.

### 2.3. Statistical Analysis

Data were analyzed with SPSS Statistics version 26.0 for Windows (SPSS, Inc., Chicago, IL, USA) and GraphPad Prism version 8.0 (Graphpad Software Inc., San Diego, CA, USA). All data were tested for Gaussian distribution. Continuous and normally distributed data are presented as mean ± standard deviation (SD), non-normally distributed data as median [interquartile range (IQR)], and categorical data as number (percentage). Differences in clinical characteristics and HSP levels between patients with and without AF were tested with independent-samples *t*-test, Mann–Whitney and Chi-square test. Differences in clinical characteristics and HSP levels between patients without a history of AF, PAF, PeAF and LSPeAF were tested with one-way analysis of variance (ANOVA), Kruskal–Wallis test, Chi-square test and Fisher’s Exact test. When serum HSP levels were below detection limit of the ELISA (only for *n* = 7 cvHSP and *n* = 41 HSP60 samples), values at the lower limit of detection were used for statistical analysis. HSP levels are not normally distributed and are Log-transformed for statistical analysis (original HSP values are presented in Tables and Figures). Uni- and multivariate linear regression was used to correlate baseline serum HSP levels with clinical parameters, and bivariate spearman correlation was used to correlate AF recurrence with clinical parameters. The difference between baseline and follow-up serum HSP levels was calculated with a repeated measures model. To relate HSP levels over time with AF recurrence and to analyze the sensitivity of the results, sensitivity analysis was performed using joint modeling. Hereto, the occurrence of the first AF recurrence (endpoint) in relation to the standardized (‘Z’) value of log2(HSP) was modeled, while using all measurements up to and including the moment of the first AF recurrence in endpoint event cases, and all measurements in those who remained event-free. A two-sided *p* value of < 0.05 indicates statistical significance.

## 3. Results

### 3.1. Study Population

The entire study population consisted of 297 patients (67% males, age 56.7 ± 13.4 years), including a control group of 98 patients without AF and a study group of 199 patients with either PAF (*n* = 86, 29%), PeAF (*n* = 108, 36.4%) or LSPeAF (*n* = 5, 1.7%) AF. Table 1 outlines baseline characteristics of the entire study population. Patients with AF were older (*p* < 0.001), more often male (*p* < 0.001), had a higher BMI (*p* < 0.001), more often had hypertension (*p* < 0.001), more often had diabetes mellitus (*p* < 0.001) and more often had dyslipidemia (*p* < 0.05), compared to patients without AF (Table 1). Clinical parameters did not differ between patients with paroxysmal and persistent AF, except for an impaired LVF (*p* < 0.001) and larger left atrial volume (*p* < 0.001) in the persistent AF patients.

### 3.2. Baseline HSP Levels Related to Clinical Stage of AF

**Figure 1:**
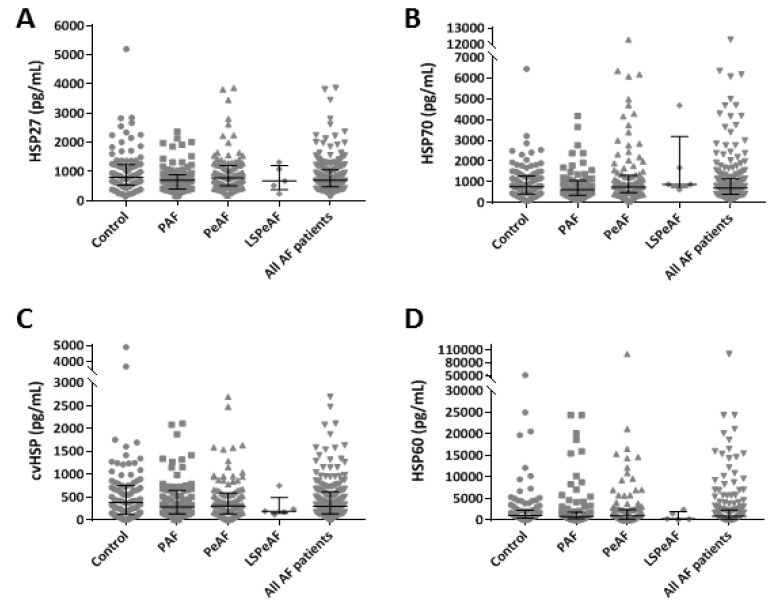
Baseline heat shock protein (HSP) serum levels in patients without and with (paroxysmal, persistent and longstanding persistent) atrial fibrillation (AF). HSP27 (**A**), HSP70 (**B**), cvHSP (**C**) and HSP60 (**D**) expression levels (pg/mL) in baseline serum of control, paroxysmal atrial fibrillation (PAF), persistent atrial fibrillation (PeAF), longstanding persistent atrial fibrillation (LSPeAF) and all AF patients.

To study the relationship between baseline HSP levels and the stage of AF, HSP27, HSP70, cvHSP and HSP60 levels were determined in serum samples of PAF, PeAF and LSPeAF patients and compared to controls. Figure 1 shows baseline concentrations of serum HSP27, HSP70, cvHSP and HSP60 of both control and AF patients; corresponding values are depicted in Supplemental Table S1. These findings and the absence of a correlation between AF stage and HSP levels after correction for potential confounders in a multivariate model (Supplemental Tables S2 and S3) indicate that there are no differences in serum HSP values between the control patients and AF patients with PAF, PeAF and LSPeAF.

### 3.3. Relation between Baseline HSP Levels and AF Recurrence

In the total AF population, AF recurrence was significantly correlated to AF stage, age, dyslipidemia and LVF, but not related to medication usage (Supplemental Table S4).

**Figure 2:**
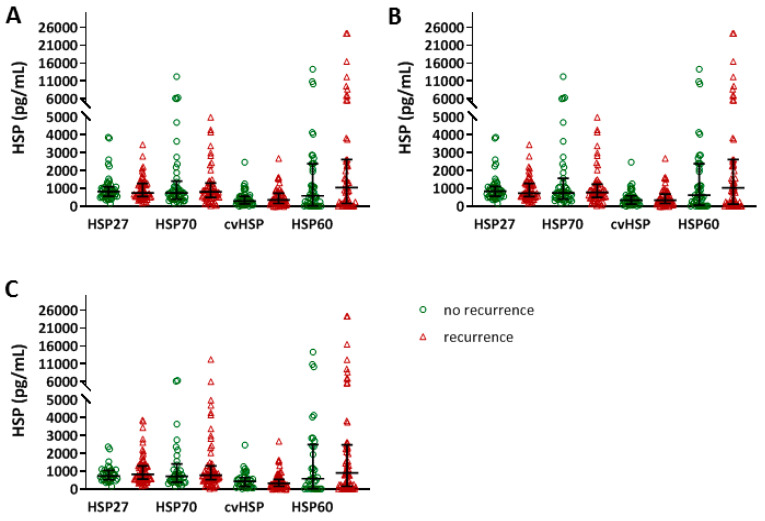
No differences in baseline HSP27, HSP70, cvHSP or HSP60 serum levels between patients with and without AF recurrence after electro cardioversion (ECV). HSP27, HSP70, cvHSP and HSP60 serum levels (pg/mL) at baseline, comparing patients with and without AF recurrence within 3 months (**A**), 6 months (**B**) and 1 year (**C**) after ECV treatment.

After ECV, AF recurrence was determined within 3 months (*n* = 52, 53.1%), 6 months (*n* = 55, 56.1%) and one year (*n* = 64, 65.3%) (Supplemental Table S5). Figure 2 shows the distribution of the various HSP levels in patients with AF recurrence (red), compared to the remainder of the ECV population (green). Baseline HSP27, HSP70, cvHSP and HSP60 levels did not differ between patients with or without AF recurrences within the first year after ECV (Figure 2, Supplemental Tables S5 and S6).

**Figure 3:**
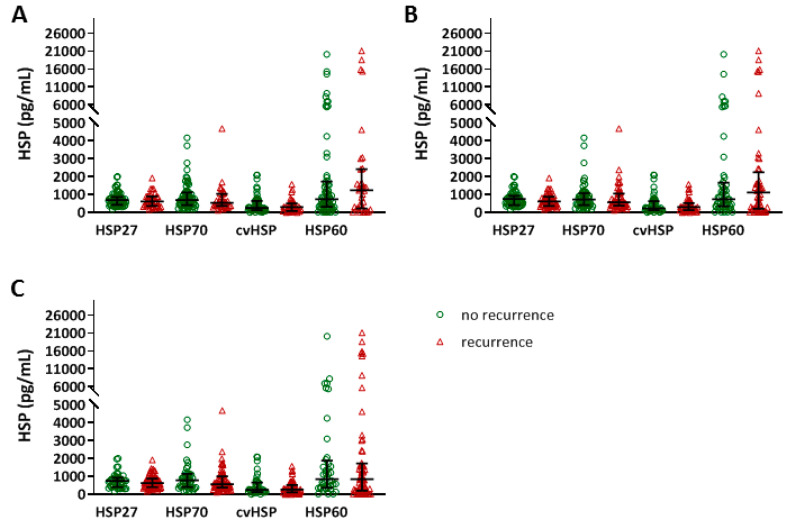
No differences in baseline HSP27, HSP70, cvHSP or HSP60 serum levels between patients with and without AF recurrence after PVI. HSP27, HSP70, cvHSP and HSP60 serum levels (pg/mL) at baseline, comparing patients with and without AF recurrence within 3 months (**A**), 6 months (**B**) and 1 year (**C**) after pulmonary vein isolation (PVI) treatment.

**Figure 4:**
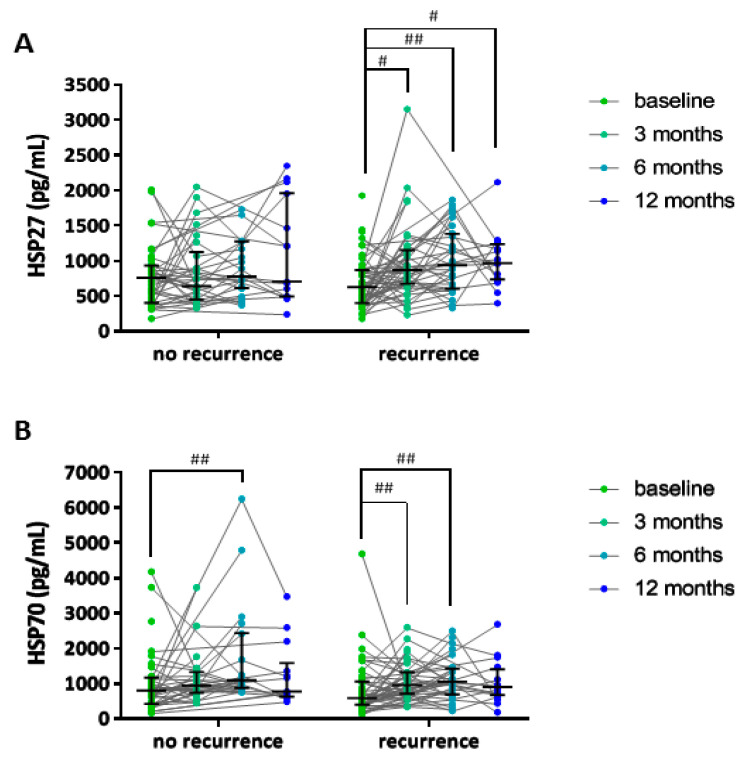
HSP27 and HSP70 levels in follow-up serum were higher than baseline in patients having AF recurrence. HSP27 serum levels (pg/mL) at baseline and at 3-, 6- and 12-months follow-up in patients having AF recurrence within one year, compared to patients not having AF recurrence (**A**). HSP70 serum levels (pg/mL) at baseline and at 3-, 6- and 12-months follow-up in patients having AF recurrence within one year, compared to patients not having AF recurrence (**B**). ^#^ *p* < 0.05 and ^##^ *p* < 0.01 compared to baseline serum HSP levels.

AF recurrences after PVI occurred within 3 months (*n* = 34, 34%), 6 months (*n* = 47, 47%) and within 1 year (*n* = 59, 58%) (Supplemental Table S7). For all time points, AF episodes were more often observed in patients with PeAF or LSPeAF, compared to patients with PAF (*p* < 0.01). Baseline serum HSP levels did not discriminate between patients with and without AF recurrence, as demonstrated in Figure 3, and Supplemental Table S6. However, HSP27 and HSP70 levels were significantly increased at 3-, 6- and 12-months post-PVI treatment in patients with an AF recurrence within one year, compared to baseline levels (Figure 4 and Supplemental Table S6). The increase in serum HSP27 levels, and not HSP70 levels, corrected for repeated measures, was significantly associated with AF recurrence (*p* < 0.013), substantiating the role of HSP27 in the prediction of AF recurrence after PVI.

To relate HSP levels over time with AF recurrence as an endpoint, joint modeling was utilized. Therefore, the occurrence of first AF recurrence (endpoint) in relation to the standardized (“Z”) value of log2(HSP) was modeled, while using all measurements up to and including the moment of the first AF recurrence in endpoint event cases, and all measurements in those who remained event-free. The results are depicted in the Table 2 (row “all events”). One standard deviation (SD) difference in log2 HSP27 level was associated with a hazard ratio (HR) of 1.32 for having an AF recurrence, supporting the outcomes from the repeated measure model. Unfortunately, the joint modeling for HSP70 did not converge, and therefore no reliable HR estimate could be obtained.

### 3.4. Sensitivity Analysis Non-Random Dropout

Although an association between HSP27 levels and the recurrence of AF after PVI was observed, sensitivity analyses were conducted to evaluate the robustness of our findings in relation to possible non-random drop out. Therefore, joint models based on the following sensitivity datasets were run: all available data (all events); patients with an AF recurrence at 3 months who also had an HSP measurement at 3 months (21 cases), in combination with patients who were free of AF recurrence at 3 months and with an HSP measurement ≥3 months (54 patients) (complete until 3 m); patients with an AF recurrence at 3 or 6 months who also had an HSP measurement at the moment of the AF recurrence (29 cases), in combination with patients who were free of AF recurrence at 6 months and with an HSP measurement ≥6 months (36 patients) (complete until 6 m); and patients with an AF recurrence at 3-, 6- or 12-months who also had an HSP measurement at the moment of the AF recurrence (31 cases), in combination with patients who were free of AF recurrence at 12 months and with an HSP measurement at 12 months (13 patients) (complete until 12 m). The findings of the sensitivity analyses are provided in Table 2. HSP27 levels were associated with an HR of 2.03 for having an AF recurrence within 6 months post PVI treatment. The results confirm that HSP27 is associated with AF recurrence, and may be used as a predictor. However, HSP70 does not seem to be associated with AF recurrence.

## 4. Discussion

In this study, we observed that serum HSP27, HSP70, cvHSP and HSP60 levels in control patients and patients with paroxysmal and (longstanding) persistent AF were comparable between the groups. Thus, HSP27, HSP70, cvHSP or HSP60 levels could not discriminate between the different AF stages in the total AF population. Additionally, at baseline, HSP27 and HSP70 serum levels were similar in patients without and with AF recurrence within one year after treatment. However, both HSP27 and HSP70 levels were higher at 3-, 6- and 12- months post-PVI compared to baseline levels in patients with AF recurrence within one year. After joint modeling and sensitivity analyses, only increased HSP27 levels post-PVI remained as a predictor of AF recurrence.

### 4.1. Heat Shock Proteins Are Not Biomarkers to Differentiate the Stage of Atrial Fibrillation

There is a great need for biomarkers to stage AF and to improve the selection of the proper treatment for patients. Several (AF-related) serum biomarkers are routinely measured in clinical practice, such as natriuretic peptide, troponin I, troponin T, creatinine and C-reactive protein [24,25,26], but lack specificity for AF. Our findings indicate no role for HSP27, HSP70, cvHSP or HSP60 as a biomarker for the presence or staging of AF. Our findings are in contrast to findings observed in other studies, as reports revealed a correlation between serum HSP levels and AF. Hu et al. revealed an association between baseline serum HSP27 levels and AF. In this study, serum HSP27 levels were reduced in paroxysmal AF and (longstanding) persistent AF patients, compared to controls in normal sinus rhythm [22]. Additionally, low serum HSP27 was associated with larger left atrial diameter, low atrial voltage (indication of fibrosis [27]) and non-pulmonary ectopies. In addition, serum HSP27 could predict sinus rhythm maintenance and low serum HSP27 correlated with a higher AF recurrence rate [22]. Our study could not confirm these findings. On the contrary, we observed an increase in follow-up serum HSP27 and HSP70 levels in patients who developed AF recurrence post-PVI. In addition, the increased HSP27 levels significantly correlate with AF recurrence. Despite this interesting observation, due to the current study design we cannot determine whether the increase in serum HSP levels is correlated with the duration of the AF episodes and whether this increase started prior or post AF recurrence.

Serum HSP70 levels were similar in control and AF patients in our study and that of others [22,28]. In line with our study, no correlation between clinical or echocardiographic variables and serum HSP70 levels was found and increased serum HSP70 levels at 6 months post-PVI correlated with AF recurrence, substantiating our findings [28].

In patients who underwent CABG, baseline serum HSP70 levels were not predictive for post-operative AF [29,30]. While no association between serum HSP60 level and occurrence of AF was found by Maan et al. [31], Cao et al. [32] described that patients undergoing mitral valve replacement with AF had higher plasma HSP60 levels than patients in sinus rhythm. Higher plasma HSP60 levels were also predictive for early (<7 days) post-operative AF [32]. Although not the protein itself, pre- and post- operative circulating anti-HSP60 antibodies are associated with post-operative AF in patients undergoing CABG [33]. In our study, serum HSP60, as well as cvHSP, did not discriminate between the stage of AF or AF recurrence. The findings indicate that for baseline serum HSP27, HSP70, cvHSP and HSP60 levels, no clear consensus exists for their use as a biomarker in AF.

### 4.2. Limitations and Future Directions: HSP in Relation to Degree of Electropathology

The current cross-sectional analysis provides information about whether HSP levels in serum samples of AF patients differ from controls in sinus rhythm. To elucidate whether HSP levels predict AF onset, detect early AF or detect progression of AF, a longitudinal study with repeated blood sampling for HSP measurements and AF testing in subjects with normal sinus rhythm is required. Our control serum HSP27 and HSP70 range is lower than the ranges described in other reports, which can be explained by differences in the analytic assays used [22,29,30,34] and due to differences in disease status of the various patient groups. The absence of difference in serum HSP levels between control and AF patients may be attributed to the clinical nature of the control group.

It is generally accepted that AF is a multifactorial disease and predisposing conditions, e.g., diabetes mellitus, hypertension and higher age were omnipresent in all our study groups, including the control group. It might be possible that the clinical variables we studied were too common and we might need to search for more AF specific parameters, such as markers of structural damage that are found to underlie atrial electrical conduction disorders, i.e., electropathology [35]. It has been suggested that the clinical classification of AF, based on ECG measurements, as presented in the guidelines, is inaccurate for AF staging because it is not related to the degree of atrial electropathology as measured by high-resolution epicardial mapping [36]. Additionally, undiagnosed, silent and/or very short-lasting AF episodes might have been overlooked in control patients and during follow-up of ECV or PVI patients by using ECG measurements. As previous studies revealed exhaustion of HSP levels in atrial tissue of persistent AF patients [18], in future research projects it is recommended to investigate human HSP levels in both serum and atrial tissue and their relationship to parameters related to AF-induced electrical and/or structural remodeling and also structural remodeling-induced (post-operative) AF [37]. Thus, a lack of correlation between HSPs and AF stage in the current study does not negate a role for HSPs as potential biomarker in AF. It is conceivable that serum HSP levels correlate with the degree of electropathology. Future studies with continuous monitoring of electrical parameters and HSP levels should elucidate such an association.

## 5. Conclusions

Serum HSP27, HSP70, cvHSP and HSP60 levels did not differentiate between AF stages and controls in sinus rhythm. Moreover, AF recurrence after ECV or PVI was not associated with baseline HSP levels. However, both HSP27 levels were increased during follow-up in patients with AF recurrence after ablative therapy and may be used as predictors. Future research directed at elucidation of an association between HSP levels and the degree of electropathology is recommended.

## Figures and Tables

**Table 1:** Clinical characteristics of the study population.

	Control	PAF	PeAF	LSPeAF	All AF Patients
*n* (%)	98 (33)	86 (29)	108 (36.4)	5 (1.7)	199 (67)
Group, *n* (%)					
Control	98 (100)	-	-	-	-
Electro cardioversion (ECV)	-	12 (14)	83 (76.9)	3 (60)	98 (49)
Pulmonary vein isolation (PVI)	-	74 (86)	25 (23.1)	2 (40)	101 (51)
Age (years), mean ± SD	48.2 ± 15.3	61.3 ± 9.5 ***	60.8 ± 10.6 ***	57.5 ± 9	60.9 ± 10.1 ***
Gender, male, *n* (%)	51 (52)	64 (74.4) **	81 (75) **	4 (80)	149 (74.9) ***
BMI (kg/m^2^), mean ± SD	25.1 ± 3.7	27.2 ± 3.8 *	28.8 ± 5.4 ***	30.4 ± 7.4	28.2 ± 4.9 ***
Hypertension, yes, *n* (%)	23 (23.5)	43 (50) ***	51 (47.2) **	3 (60)	97 (48.7) ***
Diabetes mellitus, yes, *n* (%)	5 (5.1)	10 (11.6)	15 (13.9)	1 (20)	26 (13.1) *
Dyslipidemia, yes, *n* (%)	16 (16.3)	25 (29.1)	33 (30.6)	3 (60)	61 (30.7) **
Thyroid disease, yes, *n* (%)	2 (2)	4 (4.7)	8 (7.4)	1 (20)	13 (6.5)
Left ventricular function (LVF), *n* (%)			*/^###^		
Normal	61 (79.2)	73 (84.9)	60 (58.3)	3 (60)	136 (70.1)
Mild impairment	10 (13)	9 (10.5)	29 (28.2)	2 (40)	40 (20.6)
Moderate impairment	3 (3.9)	3 (3.5)	10 (9.7)	0 (0)	13 (6.7)
Severe impairment	3 (3.9)	1 (1.2)	4 (3.9)	0 (0)	5 (2.9)
Missing ^†^	21	0	5	0	5
Left atrial volume index (mL/m^2^),median [IQR]	27.9 [21.2–39.7]	38.6 [29.3–48.4] *	47 [35.7–60.5] **/^##^	43.1 [25.9–73.6]	41.1 [31.8–54] **
Drugs, yes, *n* (%)					
Drugs total	52 (53.6)	84 (97.7) ***	104 (96.3) ***	5 (100)	193 (97) ***
ACE. ARB. AT2 antagonist	26 (26.8)	40 (46.5) *	48 (44.9) *	3 (60)	91 (46) **
Statin	17 (17.5)	32 (37.2) *	37 (34.3) *	4 (80) *	73 (36.7) ***
Antiarrhythmic drugs (AAD) total ^‡^	43 (44.3)	79 (91.9) ***	103 (95.4) ***	5 (100)	187 (94) ***
Class I AAD	5 (5.2)	31 (36.0) ***	14 (13) ^###^	1 (20)	46 (23.1) ***
Class II AAD	31 (32)	36 (41.9)	55 (50.9) *	1 (20)	92 (46.2) *
Class III AAD	6 (6.2)	42 (48.8)	55 (50.9)	2 (40)	99 (49.7) ***
Class IV AAD	3 (3.1)	4 (4.7)	7 (6.5)	5 (100)	11 (5.5)
Digoxin	0 (0)	6 (7) *	18 (16.7) ***	1 (20)	25 (12.6) ***

^†^ The percentages of LVF are valid percentages and corrected for the missing values; ^‡^ Patients may use more than one type of AAD; therefore, the sum of all classes is not equal to total; * *p* <0.05, ** *p* <0.01 and *** *p* <0.001 compared to control ^##^ *p* < 0.01 and ^###^ *p* < 0.001 comparing paroxysmal AF with persistent AF.

**Table 2:** Outcomes joint modeling of AF recurrence in relation to HSP levels, and sensitivity analyses.

	Patients		Samples		HSP27				HSP70			
	AF	AF-Free	AF	AF-Free	HR	95%CI LL	95%CI UL	*p*-Value	HR	95%CI LL	95%CI UL	*p*-Value
All events	59	41	114	99	1.32	1.71	1.54	<0.001	model does not converge			
Complete until 3 m	21	54	42	129	1.31	0.84	2.89	0.258	0.97	0.56	1.5	0.969
Complete until 6 m	29	36	63	99	2.03	1.17	3.7	<0.001	1.03	0.54	1.97	0.926
Complete until 12 m	31	13	70	34	model does not converge				model does not converge			

## Supplementary Materials

The following are available online at https://www.mdpi.com/2073-4409/9/9/2105/s1, Table S1: Concentration of HSPs in serum at baseline of patients without AF and with (paroxysmal, persistent or longstanding persistent) AF, Table S2: Univariate linear regression associations between clinical parameters and HSP levels in baseline serum, Table S3: Multivariate linear regression associations between AF, AF stage and recurrence within one year and HSP levels in baseline serum, corrected for potential confounders, Table S4: Bivariate spearman correlation between clinical parameters and recurrence within 3 months, 6 months and 12 months, Table S5: Clinical characteristics and serum HSP levels for subjects without AF and with (paroxysmal, persistent and longstanding persistent) AF in the ECV group, Table S6: Serum HSP concentrations after one year follow-up, Table S7: Clinical characteristics and serum HSP levels for subjects without AF and with (paroxysmal, persistent and longstanding persistent) AF in the PVI group.
