# Supplementary material for: Evaluating Serum Heat Shock Protein Levels as Novel Biomarkers for Atrial Fibrillation
Source: Cells. 2020 Sep 16;9(9):2105. doi: 10.3390/cells9092105 (PMC7564530; doi:10.3390/cells9092105)
Supplement: Supplementary file 1 [file cells-09-02105-s001.pdf]

cells MDPI

Supplemental Materials

Supplemental Table 1. Concentration of HSPs in serum at baseline of patients without AF and with (paroxysmal, persistent or longstanding persistent) AF.

	**Control**	**PAF**	**PeAF**	**LSPeAF**	**All AF patients**
N	98	86	108	5	199
HSP27, median [IQR]	807.9 [538.5-1240]	705.5 [406.5-898]	783.5 [519.8-1203]	679 [382-1206]	708 [479-1056] $^{P=0.14}$
HSP70, median [IQR]	758.7 [420.6-1287]	624.5 [359.8-1058]	750.5 [472.5-1306]	876 [751-3186]	701 [406-1144] $^{P=0.78}$
cvHSP, median [IQR]	381 [120.8-752.8]	286 [131-646]	301 [135-585]	189 [144-490]	293 [131-615] $^{P=0.62}$
HSP60, median [IQR]	1020 [438.5-2236]	775 [271.3-1804]	927 [165-2423]	246 [107.5-1941]	851 [219.7-2358] $^{P=0.17}$

Supplemental Table 2. Univariate linear regression associations between clinical parameters and HSP levels in baseline serum.

	HSP27	HSP70	cvHSP	HSP60
	St β	St β	St β	St β
AF	-0.087	0.016	-0.029	-0.079
Stage of AF	-0.048	0.066	-0.030	-0.084
Age (years)	-0.058	-0.028	0.080	-0.117*
Gender	0.077	0.012	0.089	0.130*
BMI (kg/m^2)	0.073	0.057	0.039	-0.098
Hypertension	-0.033	-0.053	0.027	-0.107
Diabetes mellitus	-0.009	0.022	0.167**	-0.098
Dyslipidemia	-0.043	0.042	0.076	-0.047
Thyroid disease	0.025	0.046	0.039	-0.007
Left ventricular function	0.061	0.072	0.116	0.059
ACE. ARB. AT2 antagonist	-0.061	-0.058	0.060	-0.146*
Statin	-0.030	0.076	0.053	-0.108
Class I AAD	-0.14*	-0.097	0.042	0.058
Class II AAD	0.031	-0.005	0.127*	0.002
Class III AAD	0.023	0.027	-0.110	-0.078
Class IV AAD	-0.080	-0.025	0.023	0.068
Digoxin	0.003	0.017	-0.013	-0.016
Recurrence within one year	0.046	-0.051	-0.102	0.003

Supplemental Table 3. Multivariate linear regression associations between AF, AF stage and recurrence within one year and HSP levels in baseline serum, corrected for potential confounders.

	**HSP27 St β corrected for age, gender, diabetes mellitus and Class I AAD**	**HSP70 St β corrected for age, gender and diabetes mellitus**	**cvHSP St β corrected for age, gender, diabetes mellitus and Class II AAD**	**HSP60 St β corrected for age, gender, diabetes mellitus and ACE. ARB. AT2 antagonist**
AF	-0.043	0.038	-0.108	0.024
Stage of AF	-0.009	0.097	-0.104	0.013
Recurrence within one year	0.035	-0.063	-0.151*	0.031

Supplemental Table 4. Bivariate spearman correlation between clinical parameters and recurrence within 3 months, 6 months and 12 months.

.	Recurrence within 3M	Recurrence within 6M	Recurrence within 12M
Stage of AF	0.212**	0.276**	0.190**
Age (years)	0.199**	0.144*	0.164*
Gender	0.086	0.088	0.065
BMI (kg/m²)	0.050	0.086	0.022
Hypertension	0.174*	0.067	0.092
Diabetes mellitus	0.057	-0.009	0.048
Dyslipidemia	0.152*	0.087	0.156*
Thyroid disease	0.081	-0.068	-0.069
Left ventricular function	0.123	0.214**	0.173*
ACE. ARB. AT2 antagonist	0.097	0.077	0.048
Statin	0.136	0.079	0.103
Class I AAD	-0.024	-0.111	-0.077
Class II AAD	0.072	-0.011	0.023
Class III AAD	-0.031	-0.041	-0.040
Class IV AAD	0.008	0.099	0.059
Digoxin	-0.029	-0.044	-0.042

Supplemental Table 5. Clinical characteristics and serum HSP levels for subjects without AF and with (paroxysmal, persistent and longstanding persistent) AF in the ECV group.

	Control	PAF	PeAF	LSPeAF	All AF patients
N	98	12	83	3	98
Age (years), mean ± SD	48.2 ± 15.3	62.2 ± 10.8	60.9 ± 11.2	60.3 ± 8.2	61 ± 11
Gender, male, N (%)	51 (52)	10 (83.3)	63 (75.9)	3 (100)	76 (77.6)
BMI (kg/m2), mean ± SD	25.1 ± 3.7	28.7 ± 3.0	29 ± 5.8	25.8 ± 1.6	28.9 ± 5.4
Hypertension, yes, N (%)	23 (23.5)	9 (75)	37 (44.6)	1 (33.3)	47 (48)
Diabetes mellitus, yes, N (%)	5 (5.1)	1 (8.3)	12 (14.5)	0 (0)	13 (13.3)
Dyslipidemia, yes, N (%)	16 (16.3)	5 (41.7)	23 (27.7)	1 (33.3)	29 (29.6)
Thyroid disease, yes, N (%)	2 (2)	0 (0)	7 (8.4)	1 (33.3)	8 (8.2)
Left ventricular function (LVF), N (%)					
Normal	61 (79.2)	10 (83.3)	40 (51.3)	2 (66.7)	52 (55.9)
Mild impairment	10 (13)	0 (0)	25 (32.1)	1 (33.3)	26 (28)
Moderate impairment	3 (3.9)	1 (8.3)	9 (11.5)	0 (0)	10 (10.8)
Severe impairment	3 (3.9)	1 (8.3)	4 (5.1)	0 (0)	5 (5.4)
Missing†	*21*	*0*	*5*	*0*	*5*
Left atrial volume index (ml/m2), median [IQR]	27.9 [21.2-39.7]	46 [29.5-54]	47.5 [37.5-60.8]	52 [N/A]	47 [35-60]
Cumulative recurrence, yes, N (%)					
Within 3 months	-	6 (50)	43 (51.8)	3 (100)	52 (53.1)
Within 6 months	-	7 (58.3)	45 (54.2)	3 (100)	55 (56.1)
Within 1 year	-	7 (58.3)	54 (65.1)	3 (100)	64 (65.3)
Baseline HSP serum levels, N	98	12	83	3	98
HSP27, median [IQR]	807.9 [538.5-1240]	766 [609.5-1655]	843 [561-1236]	522 [N/A]	795.5 [560-1235]
HSP70, median [IQR]	758.7 [420.6-1287]	317 [229-1127]	787 [510-1366]	873 [N/A]	774 [490-1333]
cvHSP, median [IQR]	381 [120.8-752.8]	388 [196.5-1178]	335 [150-615]	171 [N/A]	345 [164-635.8]
HSP60, median [IQR]	1020 [438.5-2236]	1713 [55.3-9843]	882 [132-2382]	246 [N/A]	849 [124-2445]

†The percentages of LVF are the valid percentage, thus corrected for the missing values

***P*<0.01 and ****P*<0.001 compared to PAF and PeAF

Statistical testing performed on cumulative recurrence and baseline HSP serum levels: Students T test on log transformed values (No AF vs All AF patients), Anova with Bonferroni corrections on the log transformed values (No AF vs PAF or No AF vs PeAF or No AF vs LSPeAF)

Supplemental Table 6. Serum HSP concentrations after one year follow up.

	No recurrence	Recurrence
**ECV patients**		
Baseline, N	34	64
HSP27, median [IQR]	737 [523.8-1059]	832.5 [569.5-1314]
HSP70, median [IQR]	727.5 [391-1409]	787 [533.8-1306]
cvHSP, median [IQR]	441.5 [153.5-648.8]	330.5 [164-553.3]
HSP60, median [IQR]	587 [32-2491]	907.5 [167-2482]
**PVI patients**		
Baseline, N	41	59
HSP27, median [IQR]	753 [398.5-925]	620 [394-862]
HSP70, median [IQR]	787 [406-1150]	566 [384-1040]
cvHSP, median [IQR]	247 [131-663]	280 [112-522]
HSP60, median [IQR]	854.5 [357-1888]	850 [206-1756]
3 months follow up, N	26	40
HSP27, median [IQR]	633 [445.5-1119]	862.5 [668-1147]$^{\#}$
HSP70, median [IQR]	922 [721.8-1312]	939 [693-1302]$^{\#\#}$
6 months follow up, N	19	32
HSP27, median [IQR]	771 [607-1270]	930 [598.5-1378]$^{\#\#}$
HSP70, median [IQR]	1079 [858-2415]$^{\#\#}$	1034 [675.3-1410]$^{*,\#\#}$
12 months follow up, N	15	16
HSP27, median [IQR]	700 [489-1957]	961.5 [730-1232]$^{\#}$
HSP70, median [IQR]	756.5 [489-1957]	883.5 [656.8-1385]

*$P<0.05$ compared to no recurrence, $^{\#}P<0.05$ and $^{\#\#}P<0.01$ compared to baseline serum

Statistical testing performed: Students T test on log transformed values (No recurrence vs Recurrence), Anova with Bonferroni corrections on the log transformed values (baseline vs follow up serum samples for HSP27 and HSP70 within the group of patients with or without recurrence)

Supplemental Table 7. Clinical characteristics and serum HSP levels for subjects without AF and with (paroxysmal, persistent and longstanding persistent) AF in the PVI group.

	Control	PAF	PeAF	LSPeAF	All AF patients
N	98	74	25	3	102
Age (years), mean ± SD	48.2 ± 15.3	61.2 ± 9.4	60.5 ± 8.3	53.3 ± 11.3	60.8 ± 9.1
Gender, male, N (%)	51 (52)	54 (73)	18 (72)	1 (50)	73 (72.3)
BMI (kg/m2), mean ± SD	25.1 ± 3.7	27 ± 3.9	28.1 ± 3.9	37.3 ± 7.5	27.4 ± 4.2
Hypertension, yes, N (%)	23 (23.5)	34 (45.9)	14 (56)	2 (100)	50 (49.5)
Diabetes mellitus, yes, N (%)	5 (5.1)	9 (12.2)	3 (12)	1 (50)	13 (12.9)
Dyslipidemia, yes, N (%)	16 (16.3)	20 (27)	10 (40)	2 (100)	32 (31.7)
Thyroid disease, yes, N (%)	2 (2)	4 (5.4)	1 (4)	0 (0)	5 (5)
Left ventricular function (LVF), N (%)					
Normal	61 (79.2)	63 (85.1)	20 (80)	1 (50)	84 (83.2)
Mild impairment	10 (13)	9 (12.2)	4 (16)	1 (50)	14 (13.9)
Moderate impairment	3 (3.9)	2 (2.7)	1 (4)	0 (0)	3 (3)
Severe impairment	3 (3.9)	0 (0)	0 (0)	0 (0)	0 (0)
Missing†	*21*	*0*	*0*	*0*	*0*
Left atrial volume index (ml/m2), median [IQR]	27.9 [21.2-39.7]	38.5 [29.1-47.6]	39.5 [29.3-60]	43.1 [N/A]	38.7 [29.8-49.5]
Cumulative recurrence, yes, N (%)					
Within 3 months	-	19 (25.7)	13 (54.2)¥¥	2 (100)¥¥	34 (34)
Within 6 months	-	29 (39.2)	16 (66.7)¥¥	2 (100)¥¥	47 (47)
Within 1 year	-	37 (50)	20 (83.3)¥¥	2 (100)¥¥	59 (58)
Baseline HSP serum levels, N	98	74	25	2	101
HSP27, median [IQR]	807.9[538.5-1240]	678.5 [393-869]*	610 [405-877]	1205 [N/A]	659 [400.5-891.5]**
HSP70, median [IQR]	758.7 [420.6-1287]	641 [396-1043]	529 [347-951]	3186[N/A]	636 [385.5-1058]
cvHSP, median [IQR]	381 [120.8-752.8]	276 [122.5-645]	224 [122-564.5]	211 [N/A]	248 [126-573]
HSP60, median [IQR]	1020 [438.5-2236]	739.5 [293.3-645]	1394 [483.3-5066]	1305 [N/A]	851 [297.3-1744]
3 months FU, N		50	15	2	67
HSP27, median [IQR]	-	823 [563.8-1194]#	784 [627-1115]	617.5 [N/A]	811 [569-1157]#

HSP70, median [IQR]	-	939 [721-1323]$^{\#\#\#}$	755 [541-1229]	1091 [N/A]	930.5 [711-1308]$^{\#\#\#}$
6 months FU, N		37	14	1	52
HSP27, median [IQR]	-	806 [601.5-1271]$^{\#}$	1138 [810.8-1659]$^{\#}$	440 [N/A]	907 [606.3-1282]$^{\#\#}$
HSP70, median [IQR]	-	1065 [822.5-1427]$^{\#\#\#}$	923 [660.8-1593]	1052 [N/A]	1059 [791.5-1434]$^{\#\#\#}$
12 months FU, N		22	8	1	31
HSP27, median [IQR]	-	961.5 [590.3-1339]$^{\#\#}$	841 [470-1240]	1021 [N/A]	959 [605-1261]$^{\#\#}$
HSP70, median [IQR]	-	924 [615.5-1415]	739 [637.3-916.5]	1748 [N/A]	804 [634.5-1385]$^{\#}$

†The percentages of LVF are the valid percentage, thus corrected for the missing values.

$^{*}P<0.05$ and $^{**}P<0.01$ compared to control, $^{¥¥}P<0.01$ PeAF and LSPeAF compared to PAF, $^{\#}P<0.05$, $^{\#\#}P<0.01$ and $^{\#\#\#}P<0.001$ compared to baseline

Statistical testing performed on cumulative recurrence and baseline HSP serum levels: Students T test on log transformed values (No AF vs All AF patients), Anova with Bonferroni corrections on the log transformed values (No AF vs PAF or No AF vs PeAF or No AF vs LSPeAF or baseline vs follow up (FU) serum samples for HSP27 and HSP70 within the group of patients with PAF, PeAF and All AF patients)
